# Artificial Human Sweat as a Novel Growth Condition for Clinically Relevant Pathogens on Hospital Surfaces

**DOI:** 10.1128/spectrum.02137-21

**Published:** 2022-03-31

**Authors:** Fergus Watson, C. William Keevil, John Chewins, Sandra A. Wilks

**Affiliations:** a School of Biological Sciences, University of Southampton, Southampton, United Kingdom; b Bioquell UK Ltd., Andover, United Kingdom; c School of Health Sciences, University of Southampton, Southampton, United Kingdom; University of Guelph

**Keywords:** hospital surfaces, biofilms, dry surface biofilms, hospital infections, human sweat

## Abstract

The emergence of biofilms on dry hospital surfaces has led to the development of numerous models designed to challenge the efficacious properties of common antimicrobial agents used in cleaning. This is in spite of limited research defining how dry surfaces are able to facilitate biofilm growth and formation in such desiccating and nutrient-deprived environments. While it is well established that the phenotypical response of biofilms is dependent on the conditions in which they are formed, most models incorporate a nutrient-enriched, hydrated environment dissimilar to the clinical setting. In this study, we piloted a novel culture medium, artificial human sweat (AHS), which is perceived to be more indicative of the nutrient sources available on hospital surfaces, particularly those in close proximity to patients. AHS was capable of sustaining the proliferation of four clinically relevant multidrug-resistant pathogens (Acinetobacter baumannii, Staphylococcus aureus, Enterococcus faecalis, and Pseudomonas aeruginosa) and achieved biofilm formation at concentration levels equivalent to those found *in situ* (average, 6.00 log_10_ CFU/cm^2^) with similar visual characteristics upon microscopy. The AHS model presented here could be used for downstream applications, including efficacy testing of hospital cleaning products, due to its resemblance to clinical biofilms on dry surfaces. This may contribute to a better understanding of the true impact these products have on surface hygiene.

**IMPORTANCE** Precise modeling of dry surface biofilms in hospitals is critical for understanding their role in hospital-acquired infection transmission and surface contamination. Using a representative culture condition which includes a nutrient source is key to developing a phenotypically accurate biofilm community. This will enable accurate laboratory testing of cleaning products and their efficacy against dry surface biofilms.

## INTRODUCTION

Healthcare-associated infections (HAIs) remain a serious threat to public health and a huge financial burden on governing bodies. Data published by the European Centre for Disease Prevention and Control report that approximately four million patients each year will acquire an HAI in Europe alone (1, 2). The situation is worsened by multidrug-resistant organisms (MDROs), which are often at the epicenter of HAI outbreaks, such as vancomycin-resistant enterococci, methicillin-resistant Staphylococcus aureus, and Acinetobacter spp (3–7). To combat this problem, healthcare facilities implement strategic infection prevention and control measures, such as hand hygiene compliance audits, isolation protection, and environmental decontamination; the last of these is often overlooked or undervalued (8). Some clinical studies have reported instances where only <50% of the environmental surfaces near the patient were sufficiently cleaned following routine practices (9–11). This inadequate removal of bioburden can result in nosocomial pathogens surviving as dry fomites for weeks or even months and acting as reservoirs for HAI transmission (12–14). Passaretti et al. (15) were able to demonstrate that the risk of acquiring an MDRO for newly admitted patients increases when the previous occupant of the room was a known harborer.

Much of the literature surrounding the benefits of cleaning is associated with outbreaks, and little exists focusing on the benefits of cleaning practices combatting environmental contamination in day-to-day situations (3, 8, 16–18). In contrast, systematic reviews of environmental cleaning in hospitals suggest that cleaning interventions are failing to significantly reduce acquisition levels of HAI such as Clostridium difficile and S. aureus (19–21). An estimated 65% of HAI are associated with hydrated biofilms on medical devices (22). However, the recent discovery of dry surface biofilms (DSB) has provided an alternative explanation for persistent HAI outbreaks. Biofilms are communities of surface-bound microbes encapsulated within a matrix of self-producing extracellular polymeric substances (EPS) and are phenotypically significantly more resistant to chemical and physical removal compared to planktonic alternatives (23). DSB harboring harmful bacteria have been identified in high-risk areas such as intensive care units (ICUs) despite stringent infection prevention measures. Until recently, efficacy testing standards governing surface disinfectants have been based upon planktonic suspension on representative surface materials (24). If found in abundance, biofilms may explain how nosocomial pathogens can survive for extended periods, and why approved cleaning agents fail to combat this microbial challenge.

Since the discovery of DSB, many studies have developed models for measuring the efficacy of hospital biocides against them. For example, the CDC reactor model, drip flow reactor model, and sedimentation protocol model have all been successfully used to highlight the efficacious properties of sodium hypochlorite and hydrogen peroxide vapor (25–27). However, these studies lack the representative growth conditions of a hospital setting and crucially fall short at emulating the microbial challenge in question (28). For example, CDC reactors completely submerge the biofilm coupons into the nutrient medium during the growth phase (26). Early studies postulated that biofilm formation on dry surfaces was supported by moist microclimates and limited nutrients near the patient, as opposed to the nutrient-rich and hydrated conditions frequently used in these biofilm models (29).

Healthcare studies show that the quantity of microbial soiling on environmental surfaces is linked to the frequencies of hand-touching by patients and hospital staff (30). Soiling can enhance the attachment and formation of biofilms from transmissible microbes, as well as their tolerance to biocidal activity of disinfectants (31–33). We speculate the organic matter transferred during skin-to-surface contact would provide sufficient nutrients to support DSB formation. This article proposes a novel growth medium using constituents readily found in human sweat for *in vitro* DSB formation representative of the clinical setting. The resultant biofilms can then be used for representative downstream testing of antimicrobials such as cleaning agents.

## RESULTS

Our artificial human sweat (AHS) formulation was comprised of 17 key constituents which are found abundantly on the surface of facial skin and palms and readily transferred to environmental surfaces upon contact. Human sweat of this origin is predominantly comprised of eccrine and sebaceous gland secretions, referred to here as sweat and sebum, respectively (Tables 1 and 2). In our study, we assessed the growth performance of four clinically relevant, multidrug-resistant strains of Gram-negative and Gram-positive bacteria (Acinetobacter baumannii, Staphylococcus aureus, Enterococcus faecalis, and Pseudomonas aeruginosa) under AHS conditions in terms of CFU per unit of measure. Routine nutrient broth was used in each experimental run for comparison. These strains were grown in single-species planktonic suspensions as well as biofilm cultures on stainless steel coupons. In comparison to the initial starter cultures (10^5^ to 10^6^ CFU/mL), the population viability for all species was shown to increase, indicating actual cell growth in contrast to survival of the inoculum.

**TABLE 1:** Chemical composition of four artificial human sweat formulations, and their respective concentrations, as reported in the literature for *in vitro* testing^*a*^

Composition	Concn. (M) in sweat			
	I^*b*^	II^*c*^	III^*d*^	IV^*e*^
Salt stock solution				
Primary electrolytes				
Sodium chloride	3.1 × 10^−2^	1.08 × 10^−1^		3.9 × 10^−1^
Potassium hydrogen carbonate		2.6 × 10^−3^		2.6 × 10^−3^
Sodium phosphate anhydrous monobasic		3.93 × 10^−5^		3.93 × 10^−5^
Calcium sulphate		9.71 × 10^−4^		9.71 × 10^−4^
Nitrogenous substances				
Urea	1 × 10^−2^	2.64 × 10^−2^	1.25 × 10^−05^	4.6 × 10^−2^
Ionic constituents				
Lactic acid	1.40 × 10^−2^	1.93 × 10^−2^	3.50 × 10^−05^	5 × 10^−2^
D(+)-glucose	1.70 × 10^−4^	1.54 × 10^−3^	1.94 × 10^−05^	2.2 × 10^−3^
Pyruvate		6.34 × 10^−4^	9.10 × 10^−4^	9.10 × 10^−4^
				
Amino acid stock solution			1.11 × 10^−5^	
Serine		5.31 × 10^−3^		2.17 × 10^−3^
Alanine	3.60 × 10^−4^	5.56 × 10^−3^		5.31 × 10^−3^
Glycine	3.90 × 10^−4^	7.48 × 10^−3^		5.56 × 10^−3^

a Sweats I to III are supported by *in situ* human sweat samples; sweat IV was developed by the author, optimized through prior planktonic culture growth assessments, and based on the median values presented within the literature (data not shown). pH was adjusted using NaOH or HCl to a range of 6.5 to 7.2 (75).

b According to Stefaniak and Harvey (45).

c According to Callewaert (46).

d According to Cadd et al. (47).

e Developed by author.

**TABLE 2:** Chemical composition of human sebum^*a*^

Composition	Concn. in sebum				
	I^*b*^ (% w/w)	II^*c*^ (% w/w)	III^*d*^ (v/v)	IV^*e*^ (quant. data/FM)	V^*f*^ (% w/w)
Fat stock solution					
Fatty acids	28.3			37.6%	
Palmitic acid			3.772		20.125
Myristic acid			0.656		3.500
Stearic acid			0.820		4.380
Oleic acid		17.00			
Cholesterol	4.0		0.8	3.8% or 1,032 ng	4.000
Squalene	10.60	12.40	2.00	14.6% or 28–5,311 ng	10.000
Triglycerides				21%	
Triolein	32.5	44.60			
Olive oil					33.000
Wax esters	25.0			25%	
Tristearin					
Lanolin oil					25.000
Jojoba oil		25.00			
Vitamin					
Vitamin E	trace	1.00			

a Sebums I to IV are supported by *in situ* samples of sebaceous secretion and fingerprint residue; sebum V was developed by the author, optimized through prior planktonic culture growth assessments, and based on the median values presented within the literature (data not shown). Quant., quantitative; FM, finger-mark.

b Modified from Stefaniak et al. (48).

c According to Wertz (49).

d According to Callewaert (46).

e According to Girod et al. (50).

f Developed by author.

### Planktonic growth under AHS and nutrient broth medium.

Analysis was first conducted using planktonic cultures incubated at room temperature under continuous shaking. After 5 days of incubation in AHS and nutrient broth, neither suspension displayed a statistical difference in overall viability (*P* > 0.2704); AHS averaged 7.20 ± 0.96 log_10_ CFU/mL while nutrient broth measured 7.22 ± 0.18 log_10_ CFU/mL (Table 3). In spite of this, AHS reported significant growth increases for the Gram-negative species A. baumannii and P. aeruginosa in comparison to the Gram-positive species S. aureus and E. faecalis (*P* < 0.0370). No such difference was observed for our nutrient-rich conditions.

**TABLE 3:** Descriptive statistics on the planktonic growth of four bacterial species in AHS and nutrient broth medium after 5 days incubation^*a*^

Species	Artificial human sweat		Nutrient broth	
	Mean (log_10_ CFU/mL)	SD	Mean (log_10_ CFU/mL)	SD
A. baumannii	7.97	0.80	7.23	0.58
S. aureus	6.07	0.63	7.06	0.36
E. faecalis	6.75	0.27	7.14	0.58
P. aeruginosa	8.04	0.79	7.47	0.55

a A statistical difference was observed between P. aeruginosa and, S. aureus, and E. faecalis (*P* = 0.0370 and *P* < 0.0001, respectively); and between A. baumannii and S. aureus (*P* = 0.0008). SD, standard deviation.

### Biofilm formation under AHS and nutrient broth medium.

Biofilm cultures were subject to identical starter cultures and environmental conditions, with the exception of a reduced shaking velocity (50 to 60 rpm), allowing biofilm formation in accordance with Azeredo et al. (34). The amount of biofilm per coupon after 5 days averaged 6.00 ± 0.92 log_10_ CFU/cm^2^ for AHS and 6.85 ± 0.15 log_10_ CFU/cm^2^ for nutrient broth (Table 4). We observed a higher degree of variation in the AHS biofilms for each species; however, no statistically significant difference in cell viability was found between the two medium types (*P* > 0.9999).

**TABLE 4:** Descriptive statistics on biofilm formation of four bacterial species in AHS and nutrient broth medium^*a*^

Species	Artificial human sweat		Nutrient broth	
	Mean (log_10_ CFU/cm^2^)	SD	Mean (log_10_ CFU/cm^2^)	SD
A. baumannii	5.88	0.87	6.75	0.25
S. aureus	6.20	0.75	6.89	0.08
E. faecalis	6.08	0.68	6.89	0.06
P. aeruginosa	5.83	1.48	6.90	0.21

a Prior to enumeration, biofilm cultures were rinsed with sterile buffer solution and desiccated for up to 66 h, as previously described (26). SD, standard deviation.

### Microscopy.

**FIG 1:**
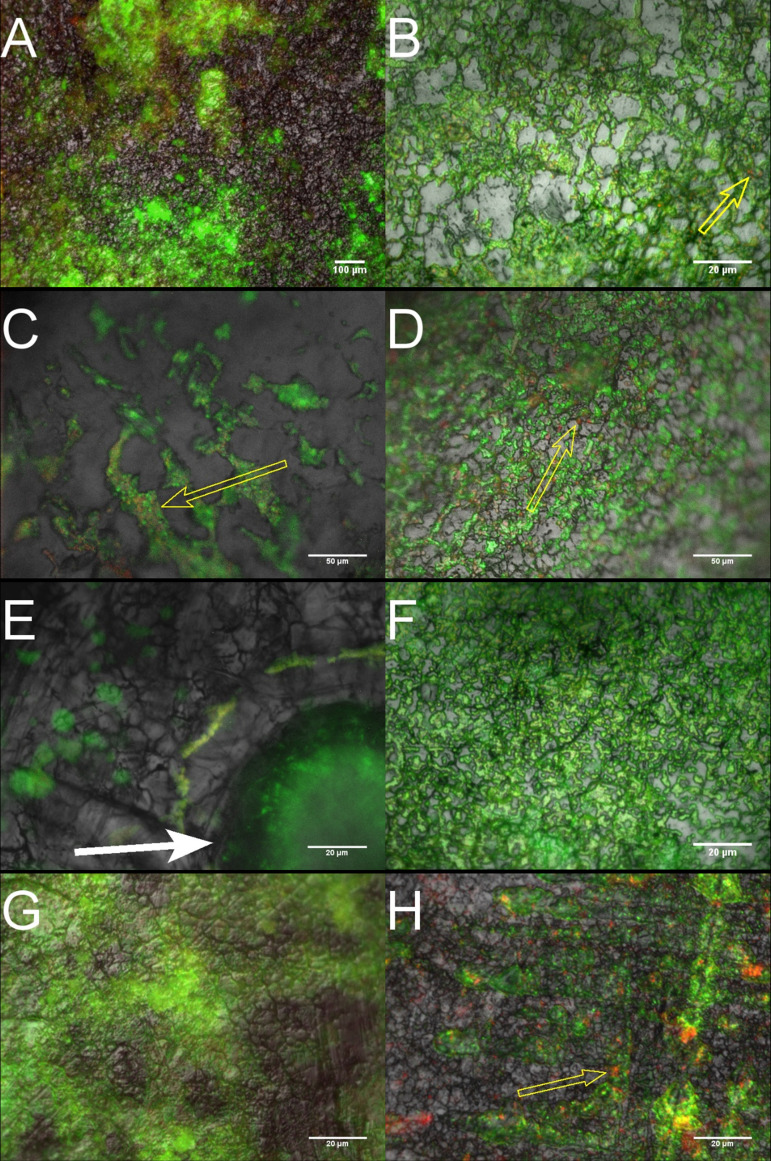
Representative EDIC/EF images of biofilm formation by A. baumannii (A and B), S. aureus (C and D), E. faecalis (E and F), and P. aeruginosa (G and H) on stainless steel coupons. Left-hand side micrographs are cultures grown under AHS; right-hand side, cultures grown in nutrient broth. The micrographs demonstrate the distinct difference in microcolony distribution between the two medium types; most notably, the spatial arrangement of colony niches around the artificial sebum aggregates, indicated by the white solid arrows. Traces of nonviable bacterial cells can be seen within the biofilms of both media types, highlighted by yellow outlined arrows.

Episcopic differential interference contrast (EDIC) microscopy combined with epifluorescence (EF) provided real-time images of our biofilm cultures. Using the bacterial viability stains SYTO-9 and propidium iodide (PI), we highlighted microcolony formation and cell aggregation along the cracks and crevices of our stainless steel coupons, as previously seen (26, 35). A mixture of viable bacteria, stained green by SYTO-9, and nonviable bacteria, stained red by PI, in both growth conditions was indicative of nutrient and oxygen gradients forming micro-niches within the biofilm matrix. Comparing micrographs of the growth conditions, it is evident that under nutrient-enriched conditions, biofilm formation is more homogenous across the surface, while AHS biofilm formation has a more heterogenous response (Fig. 1). This formation contrast is less apparent during microscopy of P. aeruginosa; this is common in Pseudomonas sp. biofilms, which exhibit confluent growth across numerous nutrient media platforms (22, 26).

## DISCUSSION

It is estimated that 20 to 40% of HAI-causing pathogens originate from cross-contamination by hospital staff and the built environment (36, 37). Since their initial discovery, DSB have been identified on a variety of surfaces in healthcare settings, including high-touch surfaces such as sanitation stations and patient furniture (38). Despite the arid nature of most clinical surfaces, sufficient moisture and nutrients must be present to support biofilm formation (29, 39–41). The risk associated between DSB abundance and patient health has yet to be determined; however, the prevalence of MDROs residing within these DSB during ongoing terminal cleaning raises considerable concern (27). Of the reported instances, *in situ* DSB range from 2.62 to 7.20 log_10_ bacteria/cm^2^ and average 5.74 log_10_ bacteria/cm^2^, on par with those formed under AHS growth conditions (6.00 log_10_ CFU/cm^2^) (42).

Synthetic sweat solutions are commonly used in the textile, cosmetics, and pharmaceuticals industries for evaluating material and chemical compatibility (43, 44). These are often minimalistic formulations and are unsupported by microbial growth studies. Our formulation was chosen to resemble the sweat originating from human facial skin or palms. The concentration values for each constituent used here are extrapolations of previously published formulations with direct comparison to *in situ* skin and fingerprint residue samples (45–51). Planktonic growth using AHS revealed a significant difference in growth response for Gram-negative and Gram-positive species. This may be explained, but not exclusively, by the different metabolic pathways employed by each cell wall type. Heterotrophic bacteria depend upon metabolism of organic sources regulated by a group of enzymes referred to as the phosphotransferase system (PTS). Unlike Gram-negative bacteria, Gram-positive bacteria lack a distinct type of PTS, which may hinder or stunt initial growth in AHS (52, 53). No such difference was observed during biofilm culture work. Gram-negative bacteria make up two-thirds of the ESKAPE pathogens, an acronym coined by the Infectious Diseases Society of America for the top antibiotic-resistant species across the globe (54). In recent years, Gram-negative bacteria have developed serious antibiotic resistance, in part due to the ease at which they exchange genetic material, a well-known trait of biofilms (55). Although not conclusive, it is plausible that the growth conditions suggested here could be enhancing the growth of Gram-negative bacteria *in situ*.

Environmental stresses such as nutrient deficiency can prompt a survival response in biofilms, reducing their susceptibility to physical and chemical removal. Studies have shown that shifts in carbon sources can impact a biofilm’s structural build. For example, Pseudomonas spp. exposed to increased amino acid concentrations can promote flat and uniform biofilm formation, while glucose can encourage larger aggregations of cells and EPS (56, 57). Our fluorescence microscopy revealed the latter for growth under AHS, with a greater heterogeneity in biofilm formation and sparse areas of densely packed colonies, whereas the nutrient broth demonstrated greater uniformity, with an even distribution of colonies across the surface. This uniformity can be seen in other studies on DSB using similar concentrations of nutrient broth (25, 26). The correct use of medium is a key influence in biofilm formation which dramatically impacts the resultant phenotypic resistance; for example, the *in vitro* antimicrobial resistance of Escherichia coli can vary between nutrient-rich and nutrient-poor medium (58). Nutrient broth contains tryptone, a rich source of amino acids; the opposite is true for AHS. These features are important when emulating *in situ* microbial challenges for biocide efficacy testing. For example, dense biofilm matrices hinder the performance of hospital disinfectants by quenching their biocidal activity and reducing their overall depth of penetration (59). As a result, microcolonies are either occluded or exposed to suboptimal levels of disinfection.

A fraction of microcolonies within the samples were stained red by PI, indicating a population of nonviable, or dead, bacteria. Johani et al. (40) reported similar staining for dead bacteria on *in situ* DSB samples from a chair at a nurses’ station. Nonviable bacteria are commonly situated at the base of the biofilm matrix and represent a population of reduced or non-metabolically active colonies (34). We suspect this is a result of nutrient and/or oxygen deprived micro-niches forming (60–62). A larger proportion of dead cells were observed under AHS conditions compared with in nutrient broth, and this is potentially reflected in our viability counts.

EPS, a major component within the matrix of a biofilm, presents similar penetrative challenges for antimicrobial agents. EPS is comprised of polysaccharides, liposaccharides, proteins, and extracellular nucleic acids (eNA). It serves as a polymer scaffolding for microorganisms within the biofilm, providing a protective barrier to chemical or physical stress. The porous structure retains key nutrients, eNA, and proteins necessary for the transfer of genetic material, such as drug resistance mechanisms (63). Biofilm cultures grown under AHS conditions observed a greater fluorescence response for PI, a red fluorophore, in comparison to those grown in nutrient broth. Recent studies have shown this may signify an increase in EPS production, or eNA secretion, rather than a loss in membrane integrity from matrix-bound colonies (64). For example, Giao et al. (65) documented that some Listeria monocytogenes biofilms grown in tap water which stained red were in fact culturable and thus viable. The group suspected the increased fluorescence was caused by eNA. Increased EPS production is often a phenotypic response to environmental stresses such as desiccation and can have a quenching effect on disinfectants, reducing efficacious properties (35).

This study was able to demonstrate a novel growth medium for modeling clinically relevant DSB for downstream efficacy testing of hospital disinfectants. Here, we generated robust single-species biofilms using four of the six ESKAPE pathogens (54). Previous examples of *in situ* clinical biofilms highlight a large diversity of species present, with each hospital, and each ward, being a unique microbiome (25). Future reiterations of our work should look to incorporate a mixed-species model using species linked to patient skin and environmental flora, as noted in genetic studies (66). For example, *Bacillus* spp. are dominant in most hospital settings and are believed to provide high levels of protection to other pathogens against disinfectant and environmental stresses (67, 68).

Our AHS formulation is adapted from previously published formulas on human sweat and fingerprint residue, and maintained a pH of 6.5 to 7.2, indicative of real life samples. There are many factors which influence the chemical composition of human sweat, including but not limited to age, sex, diet, morbidity, and environmental conditions, as well as location on the body (30, 44). Also, fingerprint residue is known to vary and degrade depending on environmental conditions, time since deposition, and substrate material (50, 69). We opted to use extrapolated concentration values of constituents commonly associated with the facial skin and palms. By using an inert substrate such as stainless steel 316, we aimed to minimize the impact on the chemical composition while maintaining a surface material representative of the hospital environment.

Despite a growing weight of evidence for DSB abundance, there is a distinct lack of *in situ* data for biofilm composition. However, limited early indications suggest a bias toward protein as a principal EPS component (25, 35, 42). We chose to use an adaptation of the sedimentation protocol for its repeatability and adaptability to nonstandard growth medium. As a result, the model lacks the physical and chemical stresses hospital surfaces may experience. Comparing the model and growth medium’s likeness to clinical DSBs this study would benefit from quantitative analyses of EPS composition for glycoconjugates, proteins, and nucleic acids.

Here, we were unable to characterize the chemical stability of our formula, as seen in other studies, due to study constraints. For example, Harvey et al. (70) analyzed the concentrations of sodium, alanine, and urea constituents among others to show less than 10% deviation over 28 days from their initial measurement. To minimize the impact of degradation, fresh solutions of AHS were generated prior to each experiment and stored for less than 24 h. We acknowledge that a variation in nutrient concentrations may be used to explain the deviations shown in biofilm cultures for AHS compared to those in nutrient broth. The standard deviation (SD) observed in biofilm culture viability for nutrient broth (average SD, 0.15) was notably lower than those of AHS (average SD, 0.95). Nonetheless our values for AHS and nutrient broth remain within the tolerance of previously accepted biofilm models (71).

Our study provides evidence for the use of low-cost, nonstandard growth medium for generating complex DSB, using a variety of MDRO associated with HAIs. AHS sustained viable cultures indicative of the target environment as well as those utilized during efficacy testing for hospital disinfectants (72). This type of testing is centered upon planktonic suspensions and there are currently no standardized testing methods against biofilms in Europe and, while protocols published by the United States are available, they are focused on hydrated biofilms (73, 74). Current alternatives for developing DSB for treatment testing using antimicrobial agents incorporate a continuous supply of nutrient-enriched medium. Although standardized sweat formulations (e.g., BS EN1811-1999) do exist for use in the pharmaceutical and textile industries, by opting for a more representative medium, as shown here, we can contribute to improving our understanding of how DSB form in hospitals and ultimately how to determine whether our infection prevention measure will work (46).

## MATERIALS AND METHODS

### Bacterial strains.

The bacterial strains used in this study possessed genes capable of expressing drug resistance mechanisms to the extended-spectrum beta-lactamase (ESBL) antibiotic group. The following strains were used: A. baumannii (NCTC: 13301), P. aeruginosa (ATCC: 15692), S. aureus (NCTC: 11939), and E. faecalis (NCTC: 13379). All species used were chosen for their ability to form biofilms and known persistence on health care surfaces.

All bacterial strains were subcultured into 10 mL NB (Southern Group Laboratory, United Kingdom), comprised of enzymatic digest of gelatin and beef extract, overnight at 37°C. The number of CFU per mL of bacterial suspension was quantified using serial dilutions and incubation on tryptic soya agar (TSA) (Sigma-Aldrich, United Kingdom) for 24 h at 37°C.

### Preparation of artificial human sweat.

All chemicals used for our artificial human sweat were ACS reagent grade and purchased from Fisher Scientific (United Kingdom) unless stated otherwise. All amino acid and salt stock solutions were prepared identically, in accordance with Harvey et al. and Calleweart (46, 70). In brief, amino acid and salt stocks were prepared in 500 mL of sterile deionized water warmed to approximately 60°C under continuous stirring. Median values of primary electrolytes, nitrogenous substances, ionic constituents, and amino acids were added to the flask in sequential order. The solution was stirred for up to 1 h to ensure no visible particulates could be seen, after which the pH was adjusted to a range of 6.5 to 7.2 with NaOH or HCl, in accordance with Kulthong et al. (75). Our fat stock solutions were prepared in accordance with Spittael et al.; whereby fatty acids, squalene, triglycerides, and wax ester constituents were heated to 85°C and mixed to a homogenous oil which was heat-sterilized (76). For planktonic culture assays, all stock solutions were mixed and heated to 60°C under continuous stirring until they were required. For biofilm culture assays, 316 stainless steel coupons were soaked in the fat stock solution overnight before being placed into a 12-well plate and submersed in amino acid and salt stock solution. All solutions were manufactured and used within a 24-h period.

### Planktonic culture.

Growth of planktonic cultures was performed in universal vials containing 9 mL of AHS solution. The vials were inoculated with 1 mL of culture inoculum at a final population concentration of ∼6 log_10_ CFU/mL and incubated at room temperature (21 to 24°C) in an orbital shaker at >100 rpm for 5 days; the medium was not replenished during the incubation stage. The number of CFU per mL of bacterial suspension was quantified using serial dilutions in sterile phosphate buffer solution (PBS) (Sigma-Aldrich, United Kingdom) and incubation on TSA (Sigma-Aldrich) for 24 h at 37°C.

### Biofilm culture.

The biofilms were generated using a static well model, also referred to as sedimentation protocol, as previously described by Merritt et al. (77). In brief, as mentioned above, previously soaked sterile stainless steel coupons (1 cm^2^) were placed into a 12-well plate containing 2 mL of stock solution and inoculated with ∼6 log_10_ CFU/mL of a single bacterial strain. The plate was then placed onto an orbital shaker at 50 to 55 rpm for 5 days, incubated at room temperature (21 to 24°C); the medium was not replenished during the incubation stage. After incubation coupons were dehydrated by means of an aquatic air pump (Hailea, United Kingdom) passing air across the surface at 3 L per minute in a 0.01-m^3^ enclosure for 48 to 66 h.

The number of CFU per cm^2^ for each coupon was determined by placing the coupon into a centrifuge tube of 2 mL sterile PBS and glass beads (2 mm), and vigorously vortexed for 15 to 30 s. The results solution was then subjected to serial dilations and incubation on TSA (Sigma-Aldrich, UK) for 24 h at 37°C. The following equation was used to obtain CFU per cm^2^:

$$\mathrm{lo}g_{10}\left(\frac{\mathrm{CFU}}{cm^{2}}\right)=\mathrm{lo}g_{10}\left[\left(\frac{\text{(mean CFU/plate)}}{\text{volume of sample plated}}\right)\times \left(\frac{\text{volume scraped into}}{\text{surface area scraped}}\right)\times (\mathrm{dilution})\right]$$

### EDIC and EF microscopy.

Samples were stained with LIVE/DEAD BacLight bacterial viability kits (Invitrogen); this included both SYTO-9 (green) and propidium iodide (red). EF microscopy was used to visualize ‘live’ and ‘dead’ bacterial cells (78).

### Experimental design and statistical analysis.

All experiments were run in triplicate unless stated otherwise. All microbial counts were transformed to log_10_ CFU/cm^2^ and all statistical calculations were performed using these values. Statistical significance of data sets was evaluated with GraphPad PRISM (v. 7.04) using one-way analysis of variance. All measurements, unless stated otherwise, were performed in triplicate.

## References

[1] Albu C, Brusin C, Ciancio B. 2015. Annual epidemiological report: antimicrobial resistance and healthcare-associated infections, p 1–28. European Centre for Disease Prevention and Control (ECDC), Stockholm, Sweden.

[2] Hanczvikkel A, Tóth Á. 2018. Quantitative study about the role of environmental conditions in the survival capability of multidrug-resistant bacteria. J Infection and Public Health 11:801–806. doi:10.1016/j.jiph.2018.05.001. 29784578

[3] Dancer SJ. 2014. Controlling hospital-acquired infection: focus on the role of the environment and new technologies for decontamination. Clin Microbiol Rev 27:665–690. doi:10.1128/CMR.00020-14. 25278571 PMC4187643

[4] Dancer SJ. 2008. Importance of the environment in meticillin-resistant *Staphylococcus aureus* acquisition: the case for hospital cleaning. Lancet Infect Dis 8:101–113. doi:10.1016/S1473-3099(07)70241-4. 17974481

[5] Martínez JA, Ruthazer R, Hansjosten K, Barefoot L, Snydman DR. 2003. Role of environmental contamination as a risk factor for acquisition of vancomycin-resistant enterococci in patients treated in a medical intensive care unit. Arch Intern Med 163:1905–1912. doi:10.1001/archinte.163.16.1905. 12963563

[6] Tankovic J, Legrand P, De Gatines G, Chemineau V, Brun-Buisson C, Duval J. 1994. Characterization of a hospital outbreak of imipenem-resistant *Acinetobacter baumannii* by phenotypic and genotypic typing methods. J Clin Microbiol 32:2677–2681. doi:10.1128/jcm.32.11.2677-2681.1994. 7852555 PMC264141

[7] Green J, Wright PA, Gallimore CI, Mitchell O, Morgan-Capner P, Brown DWG. 1998. The role of environmental contamination with small round structured viruses in a hospital outbreak investigated by reverse-transcriptase polymerase chain reaction assay. J Hospital Infection 39:39–45. doi:10.1016/S0195-6701(98)90241-9. 9617683

[8] Dancer SJ. 2009. The role of environmental cleaning in the control of hospital-acquired infection. J Hosp Infect 73:378–385. doi:10.1016/j.jhin.2009.03.030. 19726106

[9] Carling PC, Bartley JM. 2010. Evaluating hygienic cleaning in health care settings: what you do not know can harm your patients. Am J Infect Control 38:S41–S50. doi:10.1016/j.ajic.2010.03.004. 20569855

[10] Carling PC, Parry MF, Bruno-Murtha LA, Dick B. 2010. Improving environmental hygiene in 27 intensive care units to decrease multidrug-resistant bacterial transmission. Crit Care Med 38:1054–1059. doi:10.1097/CCM.0b013e3181cdf705. 20081531

[11] Carling PC, Huang SS. 2013. Improving healthcare environmental cleaning and disinfection current and evolving issues. Infect Control Hosp Epidemiol 34:507–513. doi:10.1086/670222. 23571368

[12] Strausbaugh LJ, Joseph CL. 2000. The burden of infection in long-term care. Infect Control Hosp Epidemiol 21:674–679. doi:10.1086/501712. 11083186

[13] Gontjes KJ, Gibson KE, Lansing B, Cassone M, Mody L. 2020. Contamination of common area and rehabilitation gym environment with multidrug‐resistant organisms. J Am Geriatr Soc 68:478–485. doi:10.1111/jgs.16284. 31851386 PMC9190293

[14] Mitchell BG, Dancer SJ, Anderson M, Dehn E. 2015. Risk of organism acquisition from prior room occupants: a systematic review and meta-analysis. J Hosp Infect 91:211–217. doi:10.1016/j.jhin.2015.08.005. 26365827

[15] Passaretti CL, Otter JA, Reich NG, Myers J, Shepard J, Ross T, Carroll KC, Lipsett P, Perl TM. 2013. An evaluation of environmental decontamination with hydrogen peroxide vapor for reducing the risk of patient acquisition of multidrug-resistant organisms. Clinical Infectious Diseases 56:27–35. doi:10.1093/cid/cis839. 23042972

[16] Wilks M, Wilson A, Warwick S, Price E, Kennedy D, Ely A, Millar MR. 2006. Control of an outbreak of multidrug-resistant *Acinetobacter baumannii*-*calcoaceticus* colonization and infection in an intensive care unit (ICU) without closing the ICU or placing patients in isolation. Infect Control Hosp Epidemiol 27:654–658. doi:10.1086/507011. 16807837

[17] Dancer SJ. 2011. Hospital cleaning in the 21st century. Eur J Clin Microbiol Infect Dis 30:1473–1481. doi:10.1007/s10096-011-1250-x. 21499954

[18] Donskey CJ. 2013. Does improving surface cleaning and disinfection reduce health care-associated infections? Am J Infect Control 41:S12–S19. doi:10.1016/j.ajic.2012.12.010. 23465603

[19] Mitchell BG, Hall L, White N, Barnett AG, Halton K, Paterson DL, Riley TV, Gardner A, Page K, Farrington A, Gericke CA, Graves N. 2019. An environmental cleaning bundle and health-care-associated infections in hospitals (REACH): a multicentre, randomised trial. Lancet Infectious Diseases 19:410–418. doi:10.1016/S1473-3099(18)30714-X. 30858014

[20] Anderson DJ, Moehring RW, Weber DJ, Lewis SS, Chen LF, Schwab JC, Becherer P, Blocker M, Triplett PF, Knelson LP, Lokhnygina Y, Rutala WA, Sexton DJ. 2018. Effectiveness of targeted enhanced terminal room disinfection on hospital-wide acquisition and infection with multidrug-resistant organisms and *Clostridium difficile*: a secondary analysis of a multicentre cluster randomised controlled trial with crossover design (BETR Disinfection. Lancet Infect Dis 18:845–853. doi:10.1016/S1473-3099(18)30278-0. 29880301 PMC6487496

[21] Anderson DJ, Chen LF, Weber DJ, Moehring RW, Lewis SS, Triplett PF, Blocker M, Becherer P, Schwab JC, Knelson LP, Lokhnygina Y, Rutala WA, Kanamori H, Gergen MF, Sexton DJ. 2017. Enhanced terminal room disinfection and acquisition and infection caused by multidrug-resistant organisms and *Clostridium difficile* (the Benefits of Enhanced Terminal Room Disinfection study): a cluster-randomised, multicentre, crossover study. Lancet 389:805–814. doi:10.1016/S0140-6736(16)31588-4. 28104287 PMC5935446

[22] Percival S, Williams D, Cooper T, Randle J. 2014. Biofilms in infection prevention and control: a healthcare handbook, 1st ed. Elsevier, Amsterdam, the Netherlands.

[23] Webber MA, Whitehead RN, Mount M, Loman NJ, Pallen MJ, Piddock LJV. 2015. Parallel evolutionary pathways to antibiotic resistance selected by biocide exposure. J Antimicrob Chemother 70:2241–2248. doi:10.1093/jac/dkv109. 25953808 PMC4500774

[24] Gebel J, Exner M, French G, Chartier Y, Christiansen B, Gemein S, Goroncy-Bermes P, Hartemann P, Heudorf U, Kramer A, Maillard JY, Oltmanns P, Rotter M, Sonntag HG. 2013. The role of surface disinfection in infection prevention. GMS Hyg Infect Control 8:1. doi:10.3205/dgkh000210. PMC3746601 23967396

[25] Ledwoch K, Maillard J-Y. 2018. *Candida auris* dry surface biofilm (DSB) for disinfectant efficacy testing. Materials 12:18. doi:10.3390/ma12010018. PMC6337396 30577589

[26] Watson F, Keevil CW, Wilks SA, Chewins J. 2018. Modelling vaporised hydrogen peroxide efficacy against mono-species biofilms. Sci Rep 8:1–7. doi:10.1038/s41598-018-30706-0. 30115938 PMC6095907

[27] Almatroudi A, Tahir S, Hu H, Chowdhury D, Gosbell IB, Jensen SO, Whiteley GS, Deva AK, Glasbey T, Vickery K. 2018. *Staphylococcus aureus* dry-surface biofilms are more resistant to heat treatment than traditional hydrated biofilms. J Hosp Infect 98:161–167. doi:10.1016/j.jhin.2017.09.007. 28919336

[28] Buckingham-Meyer K, Goeres DM, Hamilton MA. 2007. Comparative evaluation of biofilm disinfectant efficacy tests. J Microbiol Methods 70:236–244. doi:10.1016/j.mimet.2007.04.010. 17524505

[29] Hu H, Johani K, Gosbell IB, Jacombs ASW, Almatroudi A, Whiteley GS, Deva AK, Jensen S, Vickery K. 2015. Intensive care unit environmental surfaces are contaminated by multidrug-resistant bacteria in biofilms: combined results of conventional culture, pyrosequencing, scanning electron microscopy, and confocal laser microscopy. J Hosp Infect 91:35–44. doi:10.1016/j.jhin.2015.05.016. 26187533

[30] Adams CE, Smith J, Watson V, Robertson C, Dancer SJ. 2017. Examining the association between surface bioburden and frequently touched sites in intensive care. J Hosp Infect 95:76–80. doi:10.1016/j.jhin.2016.11.002. 27912981

[31] Ali IAA, Cheung BPK, Yau JYY, Matinlinna JP, Lévesque CM, Belibasakis GN, Neelakantan P. 2020. The influence of substrate surface conditioning and biofilm age on the composition of *Enterococcus faecalis* biofilms. Int Endod J 53:53–61. doi:10.1111/iej.13202. 31408199

[32] Chowdhury D, Rahman A, Hu H, Jensen SO, Deva AK, Vickery K. 2019. Effect of disinfectant formulation and organic soil on the efficacy of oxidizing disinfectants against biofilms. J Hospital Infection 103:e33–e41. doi:10.1016/j.jhin.2018.10.019. 30391488

[33] Smith SJ, Young V, Robertson C, Dancer SJ. 2012. Where do hands go? An audit of sequential hand-touch events on a hospital ward. J Hospital Infection 80:206–211. doi:10.1016/j.jhin.2011.12.007. 22297169

[34] Azeredo J, Azevedo NF, Briandet R, Cerca N, Coenye T, Costa AR, Desvaux M, Di Bonaventura G, Hébraud M, Jaglic Z, Kačániová M, Knøchel S, Lourenço A, Mergulhão F, Meyer RL, Nychas G, Simões M, Tresse O, Sternberg C. 2017. Critical review on biofilm methods. Crit Rev Microbiol 43:313–351. doi:10.1080/1040841X.2016.1208146. 27868469

[35] Almatroudi A, Hu H, Deva A, Gosbell IB, Jacombs A, Jensen SO, Whiteley G, Glasbey T, Vickery K. 2015. A new dry-surface biofilm model: an essential tool for efficacy testing of hospital surface decontamination procedures. J Microbiol Methods 117:171–176. doi:10.1016/j.mimet.2015.08.003. 26260119

[36] Weinstein RA. 1991. Epidemiology and control of nosocomial infections in adult intensive care units. The American J Medicine 91:S179–S184. doi:10.1016/0002-9343(91)90366-6. 1928162

[37] Weinstein RA, Hota B. 2004. Contamination, disinfection, and cross-colonization: are hospital surfaces reservoirs for nosocomial infection? Clin Infect Dis 39:1182–1189. doi:10.1086/424667. 15486843 PMC7107941

[38] Ledwoch K, Dancer SJ, Otter JA, Kerr K, Roposte D, Rushton L, Weiser R, Mahenthiralingam E, Muir DD, Maillard J-Y. 2018. Beware biofilm! Dry biofilms containing bacterial pathogens on multiple healthcare surfaces; a multi-centre study. J Hosp Infect 100:e47–e56. doi:10.1016/j.jhin.2018.06.028. 30026003

[39] Costa DM, Johani K, Melo DS, Lopes LKO, Lopes Lima LKO, Tipple AFV, Hu H, Vickery K. 2019. Biofilm contamination of high‐touched surfaces in intensive care units: epidemiology and potential impacts. Lett Appl Microbiol 68:269–276. doi:10.1111/lam.13127. 30758060

[40] Johani K, Abualsaud D, Costa DM, Hu H, Whiteley G, Deva A, Vickery K. 2018. Characterization of microbial community composition, antimicrobial resistance and biofilm on intensive care surfaces. J Infect Public Health 11:418–424. doi:10.1016/j.jiph.2017.10.005. 29097104

[41] Vickery K, Deva A, Jacombs A, Allan J, Valente P, Gosbell IB. 2012. Presence of biofilm containing viable multiresistant organisms despite terminal cleaning on clinical surfaces in an intensive care unit. J Hosp Infect 80:52–55. doi:10.1016/j.jhin.2011.07.007. 21899921

[42] Almatroudi A, Gosbell IB, Hu H, Jensen SO, Espedido BA, Tahir S, Glasbey TO, Legge P, Whiteley G, Deva A, Vickery K. 2016. *Staphylococcus aureus* dry-surface biofilms are not killed by sodium hypochlorite: implications for infection control. J Hosp Infect 93:263–270. doi:10.1016/j.jhin.2016.03.020. 27140421

[43] Robinson S, Robinson AH. 1954. Chemical composition of sweat. Physiol Rev 34:202–220. doi:10.1152/physrev.1954.34.2.202. 13155186

[44] Midander K, Julander A, Kettelarij J, Lidén C. 2016. Testing in artificial sweat: is less more? Comparison of metal release in two different artificial sweat solutions. Regul Toxicol Pharmacol 81:381–386. doi:10.1016/j.yrtph.2016.09.021. 27664322

[45] Stefaniak AB, Harvey CJ. 2006. Dissolution of materials in artificial skin surface film liquids. Toxicol in Vitro 20:1265–1283. doi:10.1016/j.tiv.2006.05.011. 16860531

[46] Callewaert C, Buysschaert B, Vossen E, Fievez V, Van de Wiele T, Boon N. 2014. Artificial sweat composition to grow and sustain a mixed human axillary microbiome. J Microbiol Methods 103:6–8. doi:10.1016/j.mimet.2014.05.005. 24858451

[47] Cadd S, Islam M, Manson P, Bleay S. 2015. Fingerprint composition and aging: a literature review. Sci Justice 55:219–238. doi:10.1016/j.scijus.2015.02.004. 26087870

[48] Stefaniak AB, Harvey CJ, Virji MA, Day GA. 2010. Dissolution of cemented carbide powders in artificial sweat: implications for cobalt sensitization and contact dermatitis. J Environ Monit 12:1815–1822. doi:10.1039/c0em00269k. 20730217

[49] Wertz PW. 2009. Human synthetic sebum formulation and stability under conditions of use and storage. Int J Cosmet Sci 31:21–25. doi:10.1111/j.1468-2494.2008.00468.x. 19134124

[50] Girod A, Ramotowski R, Weyermann C. 2012. Composition of fingermark residue: a qualitative and quantitative review. Forensic Sci Int 223:10–24. doi:10.1016/j.forsciint.2012.05.018. 22727572

[51] Lu GW, Valiveti S, Spence J, Zhuang C, Robosky L, Wade K, Love A, Hu L-Y, Pole D, Mollan M. 2009. Comparison of artificial sebum with human and hamster sebum samples. Int J Pharm 367:37–43. doi:10.1016/j.ijpharm.2008.09.025. 18929636

[52] Saier MH, Jr, 1977. Bacterial phosphoenolpyruvate: sugar phosphotransferase systems: structural, functional, and evolutionary interrelationships. Bacteriol Rev 41:856–871. doi:10.1128/br.41.4.856-871.1977. 339892 PMC414030

[53] Pflüger-Grau K, Görke B. 2010. Regulatory roles of the bacterial nitrogen-related phosphotransferase system. Trends Microbiol 18:205–214. doi:10.1016/j.tim.2010.02.003. 20202847

[54] Llaca-Díaz JM, Mendoza-Olazarán S, Camacho-Ortiz A, Flores S, Garza-González E. 2012. One-year surveillance of ESKAPE pathogens in an intensive care unit of Monterrey, Mexico. Chemotherapy 58:475–481. doi:10.1159/000346352. 23548324

[55] Flemming HC, Wingender J. 2010. The biofilm matrix. Nat Rev Microbiol 8:623–633. doi:10.1038/nrmicro2415. 20676145

[56] Klausen M, Heydorn A, Ragas P, Lambertsen L, Aaes-Jørgensen A, Molin S, Tolker-Nielsen T. 2003. Biofilm formation by *Pseudomonas aeruginosa* wild type, flagella and type IV pili mutants. Mol Microbiol 48:1511–1524. doi:10.1046/j.1365-2958.2003.03525.x. 12791135

[57] Shrout JD, Chopp DL, Just CL, Hentzer M, Givskov M, Parsek MR. 2006. The impact of quorum sensing and swarming motility on *Pseudomonas aeruginosa* biofilm formation is nutritionally conditional. Mol Microbiol 62:1264–1277. doi:10.1111/j.1365-2958.2006.05421.x. 17059568

[58] Petersen A, Møller Aarestrup F, Olsen JE. 2009. The *in vitro* fitness cost of antimicrobial resistance in *Escherichia coli* varies with the growth conditions. FEMS Microbiol Lett 299:53–59. doi:10.1111/j.1574-6968.2009.01734.x. 19694815

[59] Alfa MJ. 2019. Biofilms on instruments and environmental surfaces: do they interfere with instrument reprocessing and surface disinfection? Review of the literature. American J Infection Control 47:A39–A45. doi:10.1016/j.ajic.2019.02.027. 31146849

[60] Hope CK, Clements D, Wilson M. 2002. Determining the spatial distribution of viable and nonviable bacteria in hydrated microcosm dental plaques by viability profiling. J Appl Microbiol 93:448–455. doi:10.1046/j.1365-2672.2002.01703.x. 12174043

[61] Asally M, Kittisopikul M, Rué P, Du Y, Hu Z, Çağatay T, Robinson AB, Lu H, Garcia-Ojalvo J, Süel GM. 2012. Localized cell death focuses mechanical forces during 3D patterning in a biofilm. Proc Natl Acad Sci USA 109:18891–18896. doi:10.1073/pnas.1212429109. 23012477 PMC3503208

[62] Ghosh P, Ben-Jacob E, Levine H. 2013. Modeling cell-death patterning during biofilm formation. Phys Biol 10:e066006. doi:10.1088/1478-3975/10/6/066006. 24275528

[63] Arciola CR, Campoccia D, Speziale P, Montanaro L, Costerton JW. 2012. Biofilm formation in *Staphylococcus* implant infections. A review of molecular mechanisms and implications for biofilm-resistant materials. Biomaterials 33:5967–5982. doi:10.1016/j.biomaterials.2012.05.031. 22695065

[64] Rosenberg M, Azevedo NF, Ivask A. 2019. Propidium iodide staining underestimates viability of adherent bacterial cells. Sci Rep 9:1–12. doi:10.1038/s41598-019-42906-3. 31019274 PMC6482146

[65] Gião MS, Wilks SA, Azevedo NF, Vieira MJ, Keevil CW. 2009. Validation of SYTO 9/propidium iodide uptake for rapid detection of viable but noncultivable *Legionella pneumophila*. Microb Ecol 58:56–62. doi:10.1007/s00248-008-9472-x. 19043657

[66] Lax S, Sangwan N, Smith D, Larsen P, Handley KM, Richardson M, Guyton K, Krezalek M, Shogan BD, Defazio J, Flemming I, Shakhsheer B, Weber S, Landon E, Garcia-Houchins S, Siegel J, Alverdy J, Knight R, Stephens B, Gilbert JA. 2017. Bacterial colonization and succession in a newly opened hospital. Sci Transl Med 9:eaah6500. doi:10.1126/scitranslmed.aah6500. 28539477 PMC5706123

[67] Yezli S, Otter JA. 2012. Does the discovery of biofilms on dry hospital environmental surfaces change the way we think about hospital disinfection? J Hosp Infect 81:293–294. doi:10.1016/j.jhin.2012.05.012. 22727130

[68] Bridier A, Sanchez-Vizuete MDP, Le Coq D, Aymerich S, Meylheuc T, Maillard J-Y, Thomas V, Dubois-Brissonnet F, Briandet R. 2012. Biofilms of a Bacillus subtilis hospital isolate protect *Staphylococcus aureus* from biocide action. PLoS One 7:e44506. doi:10.1371/journal.pone.0044506. 22973457 PMC3433435

[69] Archer NE, Charles Y, Elliott JA, Jickells S. 2005. Changes in the lipid composition of latent fingerprint residue with time after deposition on a surface. Forensic Sci Int 154:224–239. doi:10.1016/j.forsciint.2004.09.120. 16182971

[70] Harvey CJ, LeBouf RF, Stefaniak AB. 2010. Formulation and stability of a novel artificial human sweat under conditions of storage and use. Toxicol in Vitro 24:1790–1796. doi:10.1016/j.tiv.2010.06.016. 20599493

[71] Goeres DM, Hamilton MA, Beck NA, Buckingham-Meyer K, Hilyard JD, Loetterle LR, Lorenz LA, Walker DK, Stewart PS. 2009. A method for growing a biofilm under low shear at the air-liquid interface using the drip flow biofilm reactor. Nat Protoc 4:783–788. doi:10.1038/nprot.2009.59. 19528953

[72] British Standards Institution (BSI). 2020. Chemical disinfectants and antiseptics. Methods of airborne room disinfection by automated process. Determination of bactericidal, mycobactericidal, sporicidal, fungicidal, yeasticidal, virucidal and phagocidal activities. EN 17272:2020. BSI, London, United Kingdom. https://www.en-standard.eu/bs-en-17272-2020-chemical-disinfectants-and-antiseptics-methods-of-airborne-room-disinfection-by-automated-process-determination-of-bactericidal-mycobactericidal-sporicidal-fungicidal-yeasticidal-virucidal-and-phagocidal-activities/.

[73] Goeres DM, Walker DK, Buckingham-Meyer K, Lorenz L, Summers J, Fritz B, Goveia D, Dickerman G, Schultz J, Parker AE. 2019. Development, standardization, and validation of a biofilm efficacy test: the single tube method. J Microbiol Methods 165:105694. doi:10.1016/j.mimet.2019.105694. 31491442

[74] US Environmental Protection Agency. 2013. Standard operating procedure for single tube method for measuring disinfectant efficacy against biofilm grown in the CDC biofilm reactor. SOP no. MB-20-01. US EPA Office of Pesticide Programs, Ft. Meade, MD.

[75] Kulthong K, Srisung S, Boonpavanitchakul K, Kangwansupamonkon W, Maniratanachote R. 2010. Determination of silver nanoparticle release from antibacterial fabrics into artificial sweat. Part Fibre Toxicol 7:8. doi:10.1186/1743-8977-7-8. 20359338 PMC2861638

[76] Spittaels K-J, Coenye T. 2018. Developing an *in vitro* artificial sebum model to study *Propionibacterium acnes* biofilms. Anaerobe 49:21–29. doi:10.1016/j.anaerobe.2017.11.002. 29175428

[77] Merritt JH, Kadouri DE, O'Toole GA. 2006. Growing and analyzing static biofilms. Curr Protoc Microbiol 1:1B.1. doi:10.1002/9780471729259.mc01b01s00. PMC4568995 18770545

[78] Wilks SA, Fader MJ, Keevil CW. 2015. Novel insights into the *Proteus mirabilis* crystalline biofilm using real-time imaging. PLoS One 10:e0141711. doi:10.1371/journal.pone.0141711. 26516766 PMC4627822

